# Harm reduction behaviours and harm experiences of people who use 3,4-methylenedioxymethamphetamine (MDMA) in Aotearoa New Zealand

**DOI:** 10.1186/s12954-024-00979-y

**Published:** 2024-03-21

**Authors:** Jai Whelan, Geoff Noller, Ryan D. Ward

**Affiliations:** 1 Deparment of Psychology, The University of Otago, William James Building, Level 1, 275 Leith Walk, Ōtepoti/Dunedin, New Zealand; 2 Department of General Practice and Rural Health, Dunedin School of Medicine, The University of Otago, Ōtepoti/Dunedin, New Zealand

**Keywords:** MDMA, Survey, Drug use, Harm reduction, Harm, Aotearoa, New Zealand

## Abstract

**Background:**

3,4-Methylenedioxymethamphetamine (MDMA) is drug of high prevalence in Aotearoa New Zealand and is the primary drug analysed by legal drug checking services. We aimed to address the gap in literature pertaining to MDMA-related harm reduction behaviour and harm experiences within the country.

**Methods:**

An online survey was used to assess the harm reduction behaviours (e.g., limiting consumption, planning use, seeking information) of people who use MDMA, in addition to their use of reagent testing and the major national drug checking and harm reduction service, KnowYourStuffNZ.

**Results:**

In total, 915 people completed the survey (60.7% females, aged 18–65, *median* = 24, IQR = 20–28). Frequency of various MDMA-related harm reduction behaviours differed, although these were carried out relatively frequently by most participants. Those who reported experiencing harm (physical, psychological, spiritual, social) from MDMA, or another drug presumed to be MDMA, reported less frequent harm reduction behaviours than non-harmed consumers. Reagent testing of MDMA had been conducted by 42.3% of the sample. Approximately 27% of the sample had used KnowYourStuffNZ services. Of KnowYourStuffNZ clients, 95.9% reported learning about harm reduction, and 53.3% reported changing their behaviour because of the service. Reasons for not using the KnowYourStuffNZ service were primarily lack of availability in local area (32.8%) or at relevant events (51.8%), and lack of concern with substance quality (29.8%). MDMA harm was reported by 14.4% of the sample, whilst reported harm was more common from consumption of presumably non-MDMA substances, self-reported as being mistaken for MDMA. Harm was primarily physical or psychological. Potential MDMA dependence was apparent in 6.9% of the sample.

**Conclusions:**

The findings highlight potential targets for harm reduction education and interventions and emphasize the need for greater availability of readily accessible drug checking services in Aotearoa New Zealand.

**Supplementary Information:**

The online version contains supplementary material available at 10.1186/s12954-024-00979-y.

## Background

3,4-Methylenedioxymethamphetamine (MDMA) is a popular entactogen type drug which produces increased feelings of connection, euphoria, and stimulation [1]. Globally, past-year use of MDMA was estimated at 0.4% of the population aged 15–64 [2]. In Aotearoa New Zealand (hereafter Aotearoa), recent data from the New Zealand Health Survey indicated a past year prevalence of ecstasy or MDMA use by people aged 15 or older of 4.3% [3]. However, little is currently known about MDMA-related harm reduction behaviours or harm experiences of people who use the substance.

When compared to many commonly used drugs, MDMA is relatively safe, and has scored in the bottom third in multiple drug harms rankings [4–6], including in Aotearoa [7], where it was ranked 7th lowest in harm in a list of 23 drugs, with a harm score of 7 out of 100. However, MDMA use is not risk-free, and various types of harm are associated with MDMA, particularly at high doses, such as hyperthermia, dehydration, hyponatraemia, and seizures, or serotonin syndrome when combined with other drugs [8]. Although not common, deaths in which MDMA is a factor increased in at least four countries between 2011 and 2017 [9], with a significant portion of those involving other stimulants and/or alcohol. Recent increases in high-dose MDMA pills are also of concern, particularly in the UK and Australia [10, 11]. Compared to the early period of MDMA use, relatively recent advances in synthesis, greater access to and use of illicit drug markets (including online) and popularity of electronic dance music have changed global consumption [12, 13]. The rapid changes to illicit drug markets have also led to adulteration or replacement of MDMA with novel psychoactive substances, particularly cathinones [14]. Although some have similar risk profiles to MDMA, they can cause more harm when not known to the consumer due to different mechanisms of action and potency.

MDMA-related risks are often recognised by consumers, and various studies have highlighted protective behavioural strategies utilized by people who use MDMA to reduce risk of harm before, during and after sessions of use, including planning use, acquiring from a trusted source, use of drug checking methods, avoiding use in unfamiliar environments, pre/post-loading with supplements, and altering route of administration, among others [15–17].

Colorimetric reagent tests, which aim to identify the presence or absence of a particular substance based on a reaction between the sample and reagent composition, can be personally used by people who use MDMA [18]. The value of this checking method is dependent upon accurate procedure adherence and result interpretation, however their utility is generally limited in drug checking contexts due to their inability to reliably quantify dose and difficulties with detecting adulterants, in addition to cost for consumers [19, 20]. Recent research in Australia has shown that approximately 31% of people who regularly use MDMA had personally used a colorimetric reagent test in the last year [21], the majority for checking what was presumed to be MDMA. Colorimetric reagent tests for checking MDMA (i.e., Marquis reagent) are available in-store and online in several countries, including Aotearoa.

Drug checking services that aim to identify substances for people who use drugs have existed in some form since the 1960s [22], and are now available in some form in at least 28 countries [23]. Although services vary, they all utilise at least one form of identification technology, including colorimetric reagent tests (although not ideal as a standalone testing method due to aforementioned limitations), immunoassay test strips, or higher standard analytical technologies such as infrared or mass spectrometry [24]. In general, drug checking appears to positively influence the behaviour of people who use drugs, primarily through changes in consumption intentions, consumption behaviours, and disposal of unexpected/undesired substances [25].

KnowYourStuffNZ (KYSNZ), Aotearoa’s first drug checking organization, officially came into being in 2016 following initial drug checking efforts the year prior [26]. Although drug checking processes initially sat within a legal grey area, interim drug checking legislation was made permanent in 2021, making Aotearoa the first country to explicitly legalize drug checking. This followed the publication of government-commissioned research that found 68% of people who used KYSNZ services altered their drug-related behaviour after doing so, and 87% reported increased harm reduction knowledge [27]. Whilst Fourier-transform infrared spectroscopy (FTIR) is the primary technology used by KYSNZ and other licensed drug checking providers in Aotearoa, colorimetric reagent tests and immunoassay strips are also used. Currently, no quantitative analysis can be conducted by client-facing services. The primary substance (both presumed and actual) checked by KYSNZ is MDMA [28]. However, periods of fluctuation in the national MDMA market have led to general concern, particularly in 2020–2021 where eutylone, a synthetic cathinone associated with significant harm and deaths [29], became prevalent and was often detected in combination with MDMA [28]. Additionally, various high-dose MDMA pills have also entered Aotearoa over the years which have greater potential to harm consumers [30].

Despite significant prevalence of past-year MDMA use in Aotearoa, little research exists on the MDMA-related harm reduction behaviours of people who use the drug. Given this knowledge gap, our study aimed to examine the harm reduction strategies and knowledge of this group, including perceived protective behaviours and personal use of colorimetric reagent testing. Due to the legal availability of drug checking in Aotearoa and its relevance for MDMA harm reduction, we also sought to examine the use of the KYSNZ service. Relatedly, we aimed to explore experiences of MDMA-related harm, including that which is presumed to have occurred following consumption of MDMA, and harm from other drugs initially presumed to be MDMA but later believed to be otherwise.

## Method

Ethical approval for the study was granted by the University of Otago Ethics Committee (ET21/147). The relevant preregistration for this study and associated survey items are available on the Open Science Framework (https://osf.io/ryu8n/).

### Recruitment and participants

Participants were recruited via study advertisements that were posted and shared online by drug-related groups and the research team on Facebook™, Instagram™ and Twitter™, as well as relevant national and regional Facebook™ groups and pages (e.g., drug interest groups, buy/sell groups), Reddit, and Bluelight. Physical posters containing a QR code linked to the survey were also placed in public locations in Ōtepoti/Dunedin and Pōneke/Wellington. To meet inclusion criteria, participants had to be aged 18 years or older, have lived in Aotearoa for at least one year, and previously used MDMA at least once within the country. Upon completion, participants could be entered into the draw for 1 of 20 $100 vouchers of their choice. A subset of the sample was also recruited through the University of Otago research participation system. Individuals who met inclusion criteria and were enrolled in an undergraduate psychology paper could complete the survey for a participation credit (rather than the voucher lottery) that would contribute 0.66% towards the final grade of one psychology paper of university coursework.

### Survey design

The survey was designed and completed using Qualtrics© software (Qualtrics, Provo, USA). The survey was presented in blocks such that MDMA and other drug-related blocks of questions and measures appeared first, followed by secondary measures. The survey utilized differential display logic to present additional questions when relevant. Pilot testing of the survey estimated that the survey would take between 30 and 60 min to complete, and participants were able to return to the survey through the same survey link within a month of their last access if they did not complete in a single session. Participants who failed two or more attention checks, reported use of a fake drug (i.e., Mingnectin), or who did not complete the MDMA harm reduction section of the survey were excluded from these analyses. The survey was available to complete between late December 2021 and July 2022.

### Variables & analysis

Variables collected via this survey included MDMA use behaviours, MDMA effects and consequences, other drug history, harm reduction behaviours and a number of other psychological variables. Given the aims of this paper, only select MDMA use variables, other drug use, harm and harm reduction related variables are reported. Additionally, participants who did not report MDMA consumption the past 5 years are excluded from these analyses.

Participants were asked to report their MDMA consumption using an ordinal scale for both average MDMA dose per session (response options ranged from < 50 mg to > 2000 mg), number of MDMA use occasions (from 1 to 500+) and frequency of consumption (“more than once a week” to “less than once a year”). Participants could also respond “I don’t know” regarding average dose per session. Participants were also asked if they have made the decision to stop MDMA use, and those that indicated deciding to stop MDMA use reported their frequency of use prior to making the decision.

Questions pertaining to harm reduction behaviour frequency were answered on a 5-point Likert scale (Always, Most of the time, Sometimes, Almost Never, Never). Agreement with statements about colorimetric reagent use and the KYSNZ service were measured via a 5-point Likert scale (Strongly agree, Agree, Neither agree nor disagree, Disagree, Strongly disagree). Participants were asked about short harm reduction phrases (“start low, go slow”, a broadly promoted statement, or “crush, dab, wait”, a statement promoted by The Loop UK) referring to the exercising of caution when dosing MDMA in case of product adulterants or unknown and potentially high potency pills, and use of online pill libraries (e.g., KYSNZ pill library [30]). In addition, various questions were asked about use (or lack of use) of harm reduction service, KYSNZ (see results for detail). Participants were also asked “Have you changed your drug taking behaviour as a result of using the service of KnowYourStuffNZ?”, which had a binary response option (yes/no). Due to the nature of this question, there was no way to verify responses, and no specific drug taking behaviour changes were explored.

Within the harm subsection of the survey, participants were asked whether they had experienced harm as a result of MDMA consumption. They were also asked, “Have you ever consumed a substance that you thought was MDMA but later believed was not?”, and if the response was either “definitely yes” or “probably yes”, the follow up question “Was there an occasion where you consumed a substance that you thought was MDMA but later believed was not and the substance caused you harm?”, which when answered yes, was taken as experience of perceived non-MDMA harm that came from a mistaken presumption about the identity of the substance. The measurement of this type of harm, and MDMA harm, was self-rated on a 1–5 scale (1 = Very little harm, 3 = Moderate Harm, 5 = Severe Harm) based on participant’s worst experience. The Māori (indigenous people of Aotearoa) health model Te Whare Tapa Whā [31], informed the design of the harm questions. This model, commonly used in Aotearoa, conceptualises well-being as made up of four distinct dimensions (physical, psychological, spiritual, family), and due to their importance for Māori, these dimensions formed the basis for harm response items (physical, psychological, spiritual, social; indirectly, but due to the drug). No specific subjective experiences of harm were evaluated beyond these self-report items. The Severity of Dependence Scale (SDS) [32], which has previously been evaluated within an MDMA consuming population [33], was used to assess elevated risk and potential dependence regarding MDMA.

SPSS Statistics (Version 28, IBM) was used for all analyses. Mean scores were calculated for ratings of harm. Alpha was set at 0.05, and all tests were two sided. Nonparametric testing was carried out where assumptions of parametric statistical testing were violated. Mann-Whitney U tests were used to compare group differences in harm reduction behaviours (ordinal data) between those who did and did not report harm of either type. All analyses between females and males were based on self-reported gender identity (other genders excluded due to low numbers). Spearman’s rank correlations were also calculated, with only correlations of ≥ 0.3 interpreted as meaningful due to potential inflation of correlations through shared method variance.

Variables that predicted use of colorimetric reagent use, MDMA harm, or non-MDMA harm, were explored using logistic regression. Variables included age, gender, Māori ethnicity, student status, region of residence, MDMA use frequency, average MDMA dose per session, self-reported knowledge of harm reduction, experiences of harm, use of drug checking methods (colorimetric reagent use or KYSNZ service use) and SDS score. Analysis used complete cases. Non-binary and gender diverse people (*n* = 22) were excluded from analysis due to low numbers.

Age was discretised as the assumption of linearity related to the log odds was violated. Age was categorized using dummy variables for the groups 18–21, 22–25, 26–29, 30–35, and 36+ (reference). Regions of residence included Otago (reference), Auckland, Wellington, Canterbury, and Other, which included all others. Frequency was of use was treated as categorical, and included the categories weekly or more often, fortnightly, monthly, every 2–3 months, every 4–6 months, and once a year or less often (reference). Average dose per session was also treated as categorical and included the dummy variables < 50 mg, 51-99 mg, 101-150 mg, 151-250 mg, 251 mg-500 mg, and > 500 mg, which were compared to the reference category of 100 mg. The levels of self-reported harm reduction knowledge were compared, with the lowest level of knowledge as the reference category. All other included variables were binary.

Variables that were found at the significance level of *p* < 0.10 at the univariate level were retained in the adjusted multivariate model, as the traditional cut-off level of 0.05 may exclude important variables [34]. The goodness of fit for all models was assessed using the Hosmer and Lemeshow test [35]. Adjusted odds ratio (aOR) results are reported in-text. The findings presented are primarily exploratory and descriptive. Other findings related to hypotheses logged in the associated preregistration are reported in the supplementary.

## Results

### Sample characteristics

The total sample consisted of 915 participants aged 18 to 65 (*M* = 25.59, *median* = 24, IQR = 20–28). Within the sample, 60.7% self-identified as female (gender), 36.9% as male, and 2.4% as non-binary, genderfluid or gender non-conforming. Most participants identified as belonging to the Pākehā/New Zealand European ethnic group (89.2%), whilst 14.2% identified as Māori, and 2.2% Pasifika (see Table 1 for more detail). Individuals recruited through the University of Otago research participation system accounted for 23.4% (*n* = 214) of the sample.

**Table 1:** Sample Demographic Characteristics (*n* = 915)

Variable	Percentage (n)
Gender Identity	
Female	60.7 (555)
Male	36.9 (338)
Non-binary/Genderfluid/Gender non-conforming	2.4 (22)
Sexual Orientation	
Heterosexual	73,4 (672)
Homosexual	3.7 (34)
Bisexual	17.5 (160)
Other	5.4 (49)
Age	
18–21	36.0 (329)
22–25	28.4 (260)
26–35	27.1 (248)
36–65	8.5 (78)
Ethnicity	
Pākehā/New Zealand European	89.2 (816)
Māori	14.2 (130)
Pasifika	2.2 (20)
Asian	5.0 (46)
European	8.1 (74)
Other	4.3 (39)
Religion	
Atheist	47.3 (433)
Agnostic	21.5 (197)
Christian/Catholic	10.8 (99)
Spiritual	12.5 (114)
All Other	7.9 (72)
Region of Residence	
Auckland/Tāmaki-Makau-rau	15.5 (142)
Wellington/Te Whanganui-a-Tara	13.3 (122)
Canterbury/Waitaha	18.3 (167)
Otago/Ōtākou	38.4 (351)
Other	14.5 (133)
Student	46.8 (428)
Work Status	
Full-time	52 (476)
Part-time	14.9 (136)
Casual	11.3 (103)
Unemployed	19.3 (177)
Retired	0.2 (2)
Other	2.3 (21)
Income	
< $10,000	26.3 (241)
10,000-$19,999	15.5 (142)
20,000-$29,999	6.3 (58)
30,000-$39,999	4.4 (40)
40,000-$49,999	7.4 (68)
50,000-$59,999	11.3 (103)
60,000-$69,999	8.0 (73)
70,000-$79,999	6.3 (58)
> $80,000	14.3 (132)
Ever convicted of criminal offence	6.7 (61)
Drug-related	2.0 (18)
Disability	7.1 (65)
Lifetime Mental Disorder	32.2 (295)
Medical Condition	18.3 (167)

### MDMA & other drug use

Of the 915 participants, 87.9% reported MDMA use within the past year. Almost two thirds (64.4%) reported use of MDMA on 11 or more occasions, whilst 21% reported use on 5 or less occasions (5.5% only one occasion of use). Just under a fifth (19.1%) of the sample reported making the decision to stop MDMA use, 68.0% of those deciding in the past year. Most participants (58.9%) reported using MDMA every 2–3 months or more often. Of those that could comment on their consumed dose of MDMA per session (*n* = 773), the median was 151-200 mg. Consumption of more than half a gram of MDMA per session was reported by 6.9% of participants. Most participants (86.6%) had previously consumed powder/crystal alone, whilst 59% reported ever consuming an MDMA pill/tablet. Pill/tablet form was the most common form of consumption for 9.2% (powder/crystal in a capsule/paper = 36.9%, powder/crystal alone = 53.7%, liquid *n* = 2). For detail regarding MDMA use information, see Table S1. Experience of acute MDMA effects were also measured and are outlined in Table S1.

Life-time recreational use of alcohol (98.5%), cannabis (89.9%), caffeine (88.0%), and nicotine (including tobacco; 78.7%) was common. Life-time recreational use of other drugs such as LSD (60.5%), nitrous oxide (54.4%), ketamine (43.2%), magic mushrooms (38.7%) and prescription opioids (32.0%) was also reported. The median number of standard drinks consumed in combination with MDMA was 7–9, whilst 28.8% reported consuming 10 or more standard drinks when using MDMA. The top three drugs reported as a participant’s favourite for recreational use were MDMA (29.5%), alcohol (27.3%) and cannabis (16.3%). The five drugs most commonly consumed within the same session as MDMA were alcohol (84.9%), nicotine (including tobacco; 47.3%), cannabis (30.2%), LSD (13.8%) and nitrous oxide (13%).

### Harm reduction

#### Knowledge

The majority of the sample (68.4%) reported that the amount of personal knowledge they had about harm reduction principles and practices was a moderate/large amount (moderate = 43.6%, large = 24.8%), whilst the remainder (31.6%) reported knowing nothing/little about harm reduction (nothing = 7.3%, little = 24.4%).

Just over half the sample (51.8%) reported searching online to help identify the contents of a pill presumed to contain MDMA. Further, 62.5% of participants reported knowing and understanding the broadly promoted phrase “start low, go slow” whilst 1.9% did not know the meaning but had heard the phrase. Regarding the phrase promoted by the international harm reduction service The Loop UK, “crush, dab, wait”, 22.1% reported knowledge and understanding, whilst 3.7% did not know the meaning but had heard the phrase.

#### Behavioural strategies & colorimetric reagent use

Reported frequency of MDMA-related behavioural harm reduction strategies are presented in Table 2. In general, occurrence of harm reduction strategies skewed towards more frequent, except for caution about mixing MDMA with alcohol, which had a relatively uniform distribution. Table S3 highlights correlations between these strategies.

**Table 2:** Reported Frequency of MDMA-related Harm Reduction Strategies (*n* = 915)

Item	Frequency (%)				
	Always	Most of the time	Sometimes	Almost Never	Never
I source/buy my MDMA from a reliable and trusted person	46.0	41.1	9.0	3.4	0.5
I plan my use of MDMA in advance	37.3	42.1	14.4	3.5	2.7
I take MDMA that is offered to me by strangers	1.7	3.8	16.9	32.7	44.8
When I take MDMA, I don’t take too much so that I am always in control and aware of my surroundings	35.8	38.5	18.5	6.0	1.2
I set a limit on how much MDMA I will take in a session and do not exceed that limit	32.0	29.0	18.5	14.3	6.2
I am cautious about mixing MDMA with other stimulant drugs	38.8	26.9	20.1	9.6	4.6
I am cautious about mixing MDMA with alcohol	18.5	19.8	17.3	26.2	18.3
I space out the occasions where I use MDMA well	33.6	30.6	18.5	11.4	6.0
When I take MDMA, I wait until I am feeling the effects before taking another dose	48.6	29.4	15.6	4.0	2.3
I am confident that my MDMA is actually MDMA before I take it	32.5	41.3	16.8	7.4	2.0
Following MDMA use, I make sure to get a good amount of rest	36.3	41.2	17.3	4.2	1.1
Prior to and following MDMA use, I make sure to have a healthy amount of nutrients (food, supplements, water)	33.4	36.4	20.2	6.6	3.4
I seek out information about MDMA online when I want to know something	48.5	25.0	16.2	5.7	4.6
I seek out information about MDMA from people I know and trust when I want to know something	30.3	32.3	27.0	6.9	3.5
If a substance I thought was MDMA turns out to be another substance that I have not used, I seek out information about that substance online before deciding whether to consume it	58.6	18.3	12.7	4.7	5.8
If a substance I thought was MDMA turns out to be another substance that I have not used, I seek out information about that substance from people I know and trust before deciding whether to consume it	41.0	26.0	19.8	7.3	5.9

When asked about personally testing MDMA with a colorimetric reagent test, 42.3% of the sample had done so. Of those who had used a reagent test, 18.3% had used in the past month, 18.6% within the past three months, 37.7% in the past year, and 25.3% more than a year ago. Reported agreement regarding several statements about reagent test use is presented in Table S4. Participants who used reagent tests reported moderate levels of agreement with statements related to worry that the sample may contain multiple active substances (net agreement, 58.7%) or no MDMA (net agreement, 47.0%).

Table S5 presents the results of the multivariable logistic regression used to identify predictors of colorimetric reagent test use. Those who reported progressively higher amounts of harm reduction knowledge had higher odds of previous use of a colorimetric reagent test, with a large amount of knowledge strongly predicting use of a colorimetric reagent test (aOR = 6.79). Those who did not know their average MDMA consumption dose had reduced odds of colorimetric reagent test use (aOR = 0.38). The odds of colorimetric reagent use were also greater in those who reported experience of non-MDMA harm (aOR = 1.65), and past use of KYSNZ services (aOR = 2.58).

When participants were asked whether most of their friends that use MDMA also use drug checking methods (reagents or KYSNZ), 40.8% said yes. Further, 45.6% of the sample believed that it is normal for people who use MDMA to utilize drug checking methods.

#### KnowYourStuffNZ service use

Most participants were aware of the existence of drug checking services such as KYSNZ (90.6%), however only 29.7% of those aware had previously used their services (26.9% of total sample). Of those who used the service (*n* = 246), 16.3% had done so in the past month, 13.4% in the past three months, 46.7% in the past year, and 23.6% more than a year ago. Participants accessed the KYSNZ service at pop-up/static clinics (within cities across the country but not on event sites) (59.3%), festivals or events within 10 days of the new year (41.5%), or festivals and events outside of the new year period (30.5%). Of those who were aware of KYSNZ, two thirds (*n* = 164) of those who had used the service reported prior reagent test use, compared to 35.7% (*n* = 208) of those who had not used KYSNZ.

Self-reported drug taking behaviour change was reported by 53.3% (*n* = 131) of people who used the service. No significant association between gender (male and female) and drug taking behaviour change was detected (*X*^2^ (1, *n* = 237) = 0.02, *p* = 0.887). Of those who did not report behaviour change (*n* = 115), “I think my drug taking behaviour is safe” was the primary reason selected (88.7%), followed by “Other” (12.2%), “I have trouble controlling/changing my drug taking behaviour” (3.5%), and “I don’t care about my drug taking behaviour” (3.5%). Further, 95.9% reported learning about harm reduction principles/practices through their use of the service (23.6% learned a little, 44.3% learned a moderate amount, 28.0% learned a large amount).

Participants generally reported trust in various aspects of the KYSNZ service (Table 3), although some agreed that they worry about judgement from others using the service (22.4%) or KYSNZ workers (13.4%). When people who were aware of but had not utilized the services of KnowYourStuffNZ were asked about reasons why they had not done so (Table S6), the top three reasons were “It has never been at an event I have attended” (51.8%), “It has never been available in my area” (32.8%), and “I have never been concerned with the content/quality/purity of my substances” (29.8%).

Following a brief description of the KYSNZ service, the service was selected as their preferred drug checking method by 81.7% of participants when compared to personal use of colorimetric reagent tests. Most participants (89.7%) expressed the desire to utilize KYSNZ services in the future.

**Table 3:** Percentage Agreement with Items Concerning KnowYourStuffNZ (*n* = 246)

Item	Agreement (%)				
	Strongly Agree	Agree	Neither agree nor disagree	Disagree	Strongly disagree
I trust the individuals who test my substances	80.5	18.7	0.8	-	-
I trust the infrared spectroscopy equipment used in the testing process	80.1	17.5	1.2	1.2	-
I trust the colour changing chemical used in the testing process	58.5	34.6	5.3	1.6	-
I feel nervous/anxious about being seen walking into the service premises	11.0	26.0	18.7	24.0	20.3
I worry about being judged by other individuals using the drug checking service	5.3	17.1	14.6	31.7	31.3
I worry about being judged by the individuals working for KnowYourStuffNZ	2.0	11.4	9.8	23.2	53.7
I am confident about the substance I have after leaving the service premises	72.8	24.8	1.6	-	0.8
I am comfortable going to use the drug checking service by myself	49.6	28.9	8.5	11.4	1.6

### Harm & dependence

Experience of harm resulting from MDMA use was reported by 14.4% (*n* = 132) of the sample, with the most recent occurrence of harm happening within the past year for 43.9% of those (*n* = 58). Emergency medical treatment was sought by 3.3% of participants as a result of their MDMA use, and alcohol was also consumed on 80% of these occasions. Of those harmed, the types of harm experienced were primarily psychological (78.0%), followed by physical (55.3%), social (30.3%) and spiritual (12.9%). The mean harm scores for the worst occasion of MDMA harm of each type were 3.16, 2.58, 3.03, and 3.18 respectively (approximately equating to moderate harm).

Consumption of what was initially presumed to be MDMA, but later was believed to be another drug (hereafter non-MDMA), was reported by 64.4% of the sample (“probably yes”, 33.1%; “definitely yes”, 31.3%). Experiences of harm resulting from such consumption were reported by 28.7% of those who had consumed perceived non-MDMA (*n* = 169, 18.5% of the total sample), with the most recent occurrence of harm happening within the past year for 39.1% (*n* = 66). Of those harmed, 4.3% sought emergency medical treatment due to a harm experience, which occurred in combination with alcohol consumption 68.2% of the time. The type of harm experienced was primarily psychological (68.6%), followed by physical (62.1%), social (20.7%) and spiritual (9.5%). The mean harm scores for the worst occasion of perceived non-MDMA harm for each type were 3.16, 2.72, 3.31, and 3.19 respectively (approximately equating to moderate harm). When asked what substance(s) they believed was consumed in place of MDMA on these occasions, synthetic cathinones was reported by 78.1%, whist 21.3% selected “I don’t know”. Fifty-one (30.2%) participants provided comment about what they believed the substance to contain, of which 40 mentioned a stimulant, primarily amphetamine (“speed”) or methamphetamine.

There were significant differences in the frequency of 14 of the harm reduction strategies, between those harmed by MDMA and not harmed by MDMA (Table S7). Twelve significant differences were also found between those harmed by non-MDMA and not harmed by non-MDMA (Table S8). All significant differences showed that the harm reduction behaviours were less frequent in those who reported experience of harm, except for the item “I take MDMA that is offered to me by strangers”, which was more frequent in those who reported harm.

For those who reported experiences of harm from both MDMA and non-MDMA substances, Wilcoxon signed-rank tests showed that non-MDMA harm was rated as significantly higher regarding physical harm (*n* = 31, MDMA = 2.77; non-MDMA = 3.16; *Z* = -2.489, *p* = 0.013) but not psychological harm (*n* = 45, MDMA = 3.31; non-MDMA = 3.60; *Z* = -1.922, *p* = 0.055). No similar statistical comparisons could be made between social and spiritual harm.

Scores on the SDS ranged from 0 to 12, with a mean score of 0.82 (SD = 1.57). A score of ≥ 4 (indicating potential dependence) was calculated for 6.9% (*n* = 63). Mann-Whitney U tests comparing mean SDS scores of males (*M* = 0.84) and females (*M* = 0.78) did not detect a significant difference, (*U* = 88415.5, *p* = 0.09). A chi-square test of independence did not show a significant association between gender (males versus females) and meeting the dependence threshold, *X*^2^ (1, *n* = 893) = 1.045, *p* > 0.05.

Logistic regression modelling indicated that odds of reporting experience of MDMA harm were higher in those who reported consumption of MDMA weekly or more often when compared to those who use once a year or less often (aOR = 5.05). Those reporting average doses of 101-150 mg had reduced odds of reporting MDMA harm experience compared to those reporting an average of 100 mg (aOR = 0.39). MDMA harm was also predicted by experience of non-MDMA harm (aOR = 6.29), use of KYSNZ services (aOR = 2.50), or meeting the SDS dependence threshold (aOR = 5.22) (Table 4).

**Table 4:** Predictors of MDMA-related harm experience

	MDMA harm (*n* = 893)		Non-MDMA harm (*n* = 893)	
Effect	OR (95% CI)	aOR (95% CI)	OR (95% CI)	aOR (95% CI)
Age				
18–21 vs. 36+	1.87 (0.81–4.29)	2.38 (0.91–6.22)	0.98 (0.52–1.83)	
22–25 vs. 36+	**2.22 (0.96–5.15)** ^**a**^	2.49 (0.94–6.58)	0.94 (0.49–1.80)	
26–29 vs. 36+	1.38 (0.55–3.50)	1.51 (0.53–4.33)	0.99 (0.49–1.99)	
30–35 vs. 36+	0.88 (0.88–2.54)	1.06 (0.32–3.49)	0.80 (0.37–1.73)	
Female gender	1.21 (0.82–1.79)		1.01 (0.71–1.42)	
Māori ethnicity	0.84 (0.48–1.48)		1.03 (0.64–1.66)	
Student	1.13 (0.78–1.64)		0.90 (0.64–1.26)	
Region of residence (ref = Otago)				
Auckland	1.08 (0.61–1.89)		1.26 (0.76–2.09)	
Wellington	1.13 (0.62–2.07)		1.48 (0.87–2.51)	
Canterbury	1.11 (0.65–1.88)		1.27 (0.79–2.06)	
Other	1.23 (0.71–2.16)		1.37 (0.82–2.28)	
Frequency of MDMA use (ref = once a year or less often)				
Weekly or more often	6.92 (3.1-15.46)***	5.05 (1.93–13.24)*	2.74 (1.01–7.42)*	1.31 (0.42–4.04)
Fortnightly	1.71 (0.73–3.99	0.73 (0.25–2.11)	4.94 (2.15–11.35)***	3.25 (1.25–8.44)*
Monthly	1.54 (0.76–3.13	0.87 (0.38-2.00)	4.44 (2.13–9.28)***	3.17 (1.40–7.15)**
Every 2–3 months	1.38 (0.72–2.68)	0.75 (0.35–1.63)	3.49 (1.72–7.10)***	2.56 (1.17–5.60)*
Every 4–6 months	1.22 (0.62–2.42)	0.84 (0.39–1.82)	2.46 (1.18–5.14)*	1.95 (0.88–4.33)
Average dose per session (ref = 100 mg)				
< 50 mg	**0.33 (0.10–1.06)** ^**a**^	0.28 (0.08-1.00)	0.78 (0.26–2.32)	1.65 (0.52–5.25)
51-99 mg	0.84 (0.35–1.99)	0.84 (0.33–2.16)	1.28 (0.50–3.28)	1.60 (0.58–4.42)
101-150 mg	0.51 (0.22–1.16)	0.39 (0.16–0.96)*	1.57 (0.69–3.58)	1.65 (0.68–4.01)
151-250 mg	0.97 (0.48–1.96)	0.66 (0.30–1.46)	2.37 (1.10–5.11)*	2.15 (0.94–4.94)
251-500 mg	0.95 (0.45–2.01)	0.57 (0.24–1.39)	3.24 (1.48–7.10)**	2.89 (1.22–6.87)*
> 500 mg	1.79 (0.76–4.21)	0.68 (0.23–2.04)	1.36 (0.49–3.79)	1.19 (0.38–3.78)
“I don’t know”	0.80 (0.37–1.72)	0.81 (0.35–1.89)	1.30 (0.56–3.01)	2.16 (0.86–5.39)
Self-reported harm reduction knowledge (ref = know nothing)				
Know little	0.91 (0.43–1.91)		2.01 (0.81–4.50)	1.97 (0.75–5.18)
Know moderate amount	0.88 (0.43–1.77)		**2.31 (0.96–5.55)** ^**a**^	1.91 (0.74–4.93)
Know large amount	0.68 (0.32–1.46)		3.07 (1.25–7.53)*	2.47 (0.92–6.60)
MDMA harm	-	-	5.57 (3.73–8.32)***	6.38 (4.06–10.03)***
Non-MDMA harm	5.57 (3.73–8.32)***	6.29 (3.99–9.92)***	-	-
Use of KYSNZ	1.95 (1.32–2.89)***	2.50 (1.56–3.99)***	1.92 (1.35–2.74)***	1.13 (0.74–1.73)
Use of colorimetric reagent test	1.28 (0.88–1.86)		2.15 (1.53–3.03)***	1.60 (1.06–2.41)*
Meets dependence threshold	6.34 (3.67–10.97)***	5.22 (2.70–3.99)***	1.64 (0.90–2.99)	
**Constant**		0.05***		0.01***
**Hosmer and Lemeshow Goodness-of-fit**		0.359		0.744

Note. ^a^ < 0.10 * < 0.05, ** < 0.01, *** < 0.001. ref; reference category. All significant values are bold.

Greater odds of reporting non-MDMA harm were associated with MDMA use fortnightly (aOR = 3.25), monthly (aOR = 3.17), and every 2–3 months (aOR = 2.56) when compared to once a year or less often. An average use of 251-500 mg of MDMA per session was associated with greater odds of non-MDMA harm (aOR = 2.898), as was past experience of MDMA harm (aOR = 6.38), and use of a colorimetric reagent test (aOR = 1.60) (Table 4).

## Discussion

This study aimed to fill a gap in understanding about the harm reduction behaviours and harm experiences associated with MDMA use in Aotearoa. In general, reported MDMA consumption per session approximated the global median (200 mg) as reported by the Global Drug Survey in 2020 [36]. Polydrug use history was common, and alcohol was consumed in significant quantities, with the median number of standard drinks consumed during an MDMA session being over the cut-off used when defining binge sessions [37].

Participants reported frequently carrying out a wide range of different perceived protective behavioural strategies, including many which have previously been reported in the literature, such as sourcing MDMA from someone they trust, spacing out MDMA use and consuming healthy food or supplements [15–17]. Frequency of accepting MDMA offered by strangers was also relatively low. In general, the considerable frequency with which these harm reduction behaviours are reported to be carried out is a positive observation, and likely contributes to considerable harm reduction and/or benefit maximization regarding MDMA consumption. However, despite general advice to limit or be cautious regarding alcohol intake if consuming alongside MDMA, caution about concomitant use of these substances was reported as much less frequent in comparison to the other harm reduction behaviours examined. This is of particular concern given alcohol can exacerbate MDMA-related risk of dehydration and hyperthermia [38], and alcohol intoxication may act as a barrier to harm reduction behaviours more broadly. Given that the risk of harm from various drugs would likely increase when harm reduction behaviours are carried out less often, it is unsurprising that those who reported experiences of harm (either by MDMA or non-MDMA) generally reported harm reduction behaviours to be less frequent than those who were not harmed. Although one might expect that an experience of harm may shift someone to be more harm reduction conscious and therefore alter their behaviour, recent occurrences of harm and lower experience with MDMA may have limited our ability to detect any recent changes. This is due to our line of questioning focusing on behaviour frequency rather than on behaviour change per se.

Over half of the sample reported assessing online information for MDMA pill identification purposes, and use of online sources for MDMA or other drug information were reported as frequent, highlighting the internet as a key facilitator of harm reduction for this population, consistent with previous explorations [39]. Over half of the sample had heard of and understood the phrase “start low, go slow”, which may be due to broad use of the phrase online, or in Aotearoa-specific drug messaging [40]. Fewer participants reported understanding of the second phrase, “crush, dab, wait”, although those who did may have encountered this following the 2015 #CrushDabWait campaign conducted by The Loop UK [41], which speaks to the value of international harm reduction campaigns for other countries. To increase MDMA-related harm reduction behaviours within Aotearoa, undertaking a similar campaign could prove a relatively inexpensive and effective means for reducing the harm experienced by people who use the drug.

Our findings show that previous use of reagent testing is relatively common among people who use MDMA and aligns with international research regarding this drug checking method [16, 21, 42–44]. Despite government sanctioned drug checking availability within Aotearoa, reagent test use is still more common. However, over a fifth of those who have utilized reagents reported using a large amount of their substance for the purpose of checking. This may complicate interpretation given that any color change will occur more rapidly in the presence of larger quantities, which may reduce any chance of visualising change associated with contaminants present in a sample. Furthermore, over half of those who had used reagents also reported concern that their substance may have contained more than one active ingredient. Products which are adulterated are less likely to be accurately identified via reagent tests due to their physical properties, and thus facilitating greater use of more sophisticated drug checking methods offered by drug checking organizations [45] stands as a clear goal for greater certainty of substance content and subsequent reductions in harm. This approach can also mitigate issues associated with consumer knowledge gaps regarding reagent tests [43].

We did not expect that almost a tenth of the sample would be unaware of KYSNZ, given the considerable media and debate regarding drug checking legalization in recent years [46, 47]. Use of KYSNZ was primarily via pop-up/static clinics, although use at festivals or other events was also common. This is not surprising given that drugs are also used outside of these environments [48]. Just over half of the present sample reported changing their drug taking behaviour because of KYSNZ service use, with the majority of clients learning a moderate to large amount about harm reduction through their use of KYSNZ. These data are similar to that reported internationally [49–51], and within Aotearoa [27], highlighting the positive influence that drug checking services can have on the knowledge and behaviour of people who use drugs. Furthermore, the majority of those who did not indicate behaviour change reported that they believed their drug taking behaviour was safe, indicating that lack of change was not due to a failure of KYSNZ to have positive influence over “unsafe” client behaviour. However, we were unable to determine whether or not associated behaviour was indeed “safe” with the current dataset.

KYSNZ client attitudes regarding the service itself were generally favourable regarding service volunteers, drug checking methods, and confidence in substance identity, although over 10% of clients expressed concerns relating to judgement from volunteers of the service, others using the service, and over a third agreed that they feel nervous about being seen walking into the service premises. Indeed, drug use is widely stigmatised in Aotearoa [52] and although MDMA may not be as stigmatised as other drugs (e.g., methamphetamine), these findings clearly highlight stigma-related concerns within this population that likely causes secondary harm to some KYSNZ clients and acts as a barrier to drug checking efforts [53]. To address this, public awareness campaigns may prove useful for the reduction of stigma and aid the normalization of drug checking behaviour. The increase in the number of client-facing drug checking services since the legalization of drug checking (currently three organizations) is also likely to aid normalization and uptake. Greater consideration of alternative drug checking service provision (e.g., mail-in) and service locations may also prove useful for mitigating concern about being seen using the service.

The majority of our sample did not report use of the drug checking services of KYSNZ, with the primary reasoning relating to lack of availability, both at events and within specific areas. This may also contribute to the use of reagent checking, including within the past year, given that drug checking was legal within that time, i.e., during the study period. Furthermore, people who used KYSNZ were more likely to have used a reagent test than those who had not used KYSNZ, which may also indicate a greater general concern with carrying out drug checking. However, the significant endorsement of lack of service availability, in addition to the majority preferring drug checking services over reagent tests as a methodology, is a clear indication that more individuals wish to use the service. Therefore, more resources should be directed towards drug checking service efforts in order to facilitate harm reduction. Beyond availability, the other major reason for lack of KYSNZ use was a lack of concern regarding a substance, which is likely related trust in the source of MDMA [54].While it may be the case that significant numbers of people who use MDMA have longstanding or high trust relationships with their suppliers, Aotearoa has experienced fluctuating periods of MDMA quality, where different constituents are sold in addition to or in place of MDMA [28]. With this in mind, wider attitudinal or educational interventions may need to be explored to combat common misconceptions and reasonable caution about trust in suppliers in order to increase drug checking service use within the community of people who use drugs.

Although the total number of participants reporting MDMA or non-MDMA harm were similar, approximately twice as many individuals who reported MDMA harm also reported non-MDMA harm if they reported probable non-MDMA consumption, indicating a greater likelihood of harm following consumption of non-MDMA. Given the high levels of suspected non-MDMA consumption within the sample, the relatively low percentage of participants reporting harm resulting from that consumption is a positive finding, which may highlight the benefits of generally applied harm reduction behaviours. Further, KYSNZ service use was associated with greater odds of MDMA harm, which may be indicative of behavioural change occurring following a negative experience. Although unlikely, the inverse may also be true, where harms may have occurred following engagement with KYSNZ services, however no causality can be inferred through this dataset. Colorimetric reagent use was also associated with greater odds of non-MDMA harm experience, but not MDMA harm. This result makes intuitive sense, as reagents may be used to identify non-MDMA compounds and thus reduce harm via avoidance of consumption if detected, whilst harm arising from MDMA use cannot be due to any feature identified by a reagent test. However, perceptions of substance contents may be skewed, as reagent color change based on a sample that contains only a small amount of MDMA may mask other color changes [20], and thus individuals may be led to believe that any subsequent harm was related to use of MDMA, when in fact the substance may have predominantly contained a different drug (e.g., eutylone). Such factors highlight the importance of using more sophisticated drug checking methods when available and increasing the uptake of formal drug checking services. KYSNZ service but not reagent test use was also associated with greater odds of reporting MDMA harm, which may be reflective of harm reduction information-seeking that can be obtained through KYSNZ services, although this may also reflect underlying knowledge of the limitations of colorimetric reagent use for checking adulterated MDMA.

The findings that those who have been harmed by MDMA and non-MDMA are linked may reflect a general underlying set of behaviours that increase risk of harm to drugs more generally, particularly as MDMA is a relatively safe drug compared to other commonly used substances when use is informed [7]. Consuming MDMA in such a way that increases risk of harm (e.g., higher frequency of consumption, higher dosage) is also likely to result in harm from other adulterated or otherwise misidentified substances, particularly if these other substances are primarily synthetic cathinones, which can have higher potency than MDMA [55]. The significantly higher physical harm rating for non-MDMA substances, which were commonly presumed to have been consumed in place of MDMA by those harmed within the sample, may be explained by similar means. However, self-rated harm scores from both MDMA and non-MDMA were otherwise comparable, indicating that on the most severe occasions where harm occurred, this approximated moderate harm, although such ratings may have been limited by our 5-point response range. Interestingly, but perhaps unsurprisingly, some participants reported experiences of spiritual harm from MDMA and non-MDMA use. Although wairua (spirit) is a key facet of the Māori model of health Te Whare Tapa Whā [31], a facet that is known to be at risk of harm from drug use [7], little research has explored this with respect to MDMA. Our findings indicate that MDMA (and other substance) use can indeed lead to spiritual harm within Aotearoa and highlights the importance of quantifying and further exploring this aspect of harm regarding MDMA and other drugs in Aotearoa.

Although MDMA pills were not the most commonly reported form of MDMA consumed in our sample, it is worth noting that a sizable portion of people who have consumed pills reported consuming them in their entirety, rather than in fractions. Currently, the primary drug checking method (FTIR) used by KYSNZ (and all client-facing drug checking organizations) is unable to accurately quantify MDMA dose within pills. Although the technology required for quantitative analysis is available to Environment Science and Research (a Crown institute and licensed drug checker), this is not standard practice. Consequently, drug checking services are forced to provide rough estimates based on other data sources and information gathered at the time of checking. Given that high dose pills are known to cause significant harms and death internationally, the currently crude procedure for estimating purity and dose, combined with current pill consumption behaviour, does little to reduce concerns about potential MDMA overdose risk. Further consideration should therefore be given to the expansion of drug checking methods and/or protocols to increase the accuracy of drug checking specific to pills and subsequent sharing of this information, particularly if access and consumption of pressed pills becomes more common within Aotearoa.

In addition to specific instances of harm, the SDS was also used to identify potential dependence to MDMA within the sample. Although MDMA is not typically associated with dependence, some literature suggests that features of dependence are apparent in some people who use MDMA [33]. Little work has highlighted MDMA-specific dependence issues within Aotearoa, although in the present study, 6.9% of the sample met the threshold for potential dependence (lower than the most recent findings of 14% in Australia [56], which may indicate that some consumers of MDMA experience dependence features that may warrant harm reduction intervention. In future, brief assessment for potential dependence could be integrated with services such as KYSNZ, which could be addressed (e.g. via referral) later.

We should note that the online survey methodology used in this study may introduce selection bias through the use of a convenience sample, which can limit the representativeness and generalisability of the findings. Relatedly, we cannot guarantee the complete absence of false responding, although relatively low reimbursement (or university credit) incentive and attention checks likely reduced the likelihood of this. The significant contribution to the dataset from people residing in Ōtākou/Otago is also have influenced the data regarding consumption, given that MDMA use per capita is highest within the Southern region [57]. Furthermore, many respondents indicated that they were relatively knowledgeable about drug harm reduction principles and practices, which may have contributed to their motivation for completing the survey. This may imply that our data are not reflective of MDMA consumers generally, and the lack of participants with less harm reduction knowledge may signal that our results potentially underestimate drug harm experiences and overestimate harm reduction behaviours. However, our findings may also signal that the population of MDMA consumers is relatively informed regarding MDMA and harm reduction more generally. Given that our sample skewed towards youth (as does MDMA use [3, 58]), and a fifth of the sample had 5 or fewer experience occasions, this may also partially explain relatively low use of KYSNZ services. Additionally, drug checking itself may be facilitated by select people within a social group, with bulk purchases or multiple samples of MDMA being checked on behalf of others. However, we were unable to investigate this behaviour with our dataset.

Finally, it should also be noted that, although an attempt was made to distinguish between MDMA-specific harm, and harm from another drug that was initially believed to be MDMA, there is no way to validate these reports. Although it is possible that some MDMA consumers utilised drug checking services following consumption occurrences that resulted in harm, some experiences of harm may also have been falsely attributed to other substances due to beliefs about MDMA safety or prior positive experience with the drug.

## Conclusion

To our knowledge, this paper provides the first quantitative exploration of harm reduction practices and knowledge, and MDMA or other drug (mistaken for MDMA) harm experiences of people who use MDMA in Aotearoa. Our findings show that most consumers of MDMA are carrying out harm reduction practices relatively frequently, with notable deviations regarding cautiousness around co-consumption of alcohol, and the majority of the sample not reporting use of colorimetric reagent tests or KYSNZ drug checking services.

Additionally, this research is the first external work to explore MDMA consumer considerations of KYSNZ, and to highlight reasons for not having utilized the drug checking service. Our findings clearly demonstrate that although KYSNZ is trusted and has a generally positive impact for MDMA consumers, greater access to drug checking services is needed across Aotearoa. This is of particular concern given that harm appears to be more common if consumption of another drug occurs in place of MDMA. Expansion of drug checking methods should also be considered to account for potential increases in availability of high-dose MDMA pills.

### Electronic supplementary material

Below is the link to the electronic supplementary material.

Supplementary Material 1

## Data Availability

The datasets generated and/or analysed during the current study are not publicly available for ethical reasons but may be made available from the corresponding author on reasonable request.
