# Supplementary material for: Harm reduction behaviours and harm experiences of people who use 3,4-methylenedioxymethamphetamine (MDMA) in Aotearoa New Zealand
Source: Harm Reduct J. 2024 Mar 21;21:67. doi: 10.1186/s12954-024-00979-y (PMC10956294; doi:10.1186/s12954-024-00979-y)

**Supplementary**

A significant correlation was found between non-MDMA physical harm rating and frequency of “When I take MDMA, I don’t take too much so that I am always in control and aware of my surroundings” (*n* = 116, *r* = .340, *p* < 0.001). No other significant correlations were found between non-MDMA harm ratings of any type, and frequency of harm reduction behaviours. Figure S1 highlights the portion of the sample who reported non-MDMA harm, grouped by MDMA harm experience.

A significant correlation was found between average MDMA consumed per session and frequency of “When I take MDMA, I don’t take too much so that I am always in control and aware of my surroundings” (*n* = 773, *r* = .350, *p* < 0.001), and “I set a limit on how much MDMA I will take in a session and do not exceed that limit” (*n* = 773, *r* = .400, *p* < 0.001). No other significant correlations were found between MDMA harm ratings of any type, and frequency of harm reduction behaviours.

Several significant correlations were found between MDMA-related harm reduction behaviours (Table S3). No significant correlations of ≥0.3 were found between age and frequency of MDMA related harm reduction behaviours.

Ratings of MDMA harm did not correlate with MDMA dose per session, frequency of consumption. Physical MDMA harm ratings correlated with frequency of experiencing tight/sore jaw (*n* = 73, r = -.319, *p* = 0.006). Psychological MDMA harm ratings correlated with dehydration (*n* = 103, r = -.325, *p* < 0.001). Social MDMA harm score ratings correlated with the frequency several negative MDMA effects, including anxiety (*n* = 40, r = -.404, *p* < 0.001), nausea (*n* = 40, r = -.419, *p* < 0.001), confused thoughts (*n* = 40, r = -.440, *p* < 0.001), increased bowel movement (*n* = 40, r = -.352, *p* < 0.001), dehydration (*n* = 40, r = -.377, *p* < 0.001), intense negative emotion (*n* = 40, r = -.343, *p* < 0.001), having trouble standing or moving (*n* = 40, r = -.521, *p* < 0.001) and excessive sweating (*n* = 40, r = -.577, *p* < 0.001).

SDS scores correlated with both average consumption of MDMA consumed per session (*n* = 773, *r* = .204, *p* < 0.001) and frequency of MDMA use (*n* = 915, *r* = -0.277, *p* < 0.001), but neither were above our ≥0.3 criteria.

| **Table S1**  *MDMA use variables (n* = 915*)* | |
| --- | --- |
| Variable | Percentage (*n*) |
| MDMA form (ever consumed) |  |
| Powder/crystal in a capsule/paper | 81.6 (747) |
| Powder/crystal alone | 86.6 (792) |
| Pill/tablet | 59.0 (540) |
| Liquid | 6.3 (58) |
| MDMA form (most consumed) |  |
| Powder/crystal in a capsule/paper | 36.9 (338) |
| Powder/crystal alone | 53.7 (491) |
| Pill/tablet | 9.2 (84) |
| Liquid | 0.2 (2) |
| Most common route of administration |  |
| Oral (swallowing) | 49.4 (452) |
| Oral/sublingual/buccal (finger dipping/gumming) | 5.9 (54) |
| Nasal (snorting) | 43.9 (402) |
| Rectal (shelving/boofing) | 0.2 (2) |
| Injecting | 0.3 (3) |
| Other | 0.2 (2) |
| Average dose within pressed pill |  |
| <50mg | 6.3 (23) |
| 50-100mg | 23.9 (87) |
| 101-150mg | 46.2 (168) |
| 151-200mg | 15.1 (55) |
| 201-250mg | 6.9 (25) |
| 251-300mg | 0.8 (3) |
| >300mg | 0.8 (3) |
| Pill consumption method |  |
| Take it all at once | 44.1 (239) |
| Take it in halves | 30.6 (166) |
| Take it in quarters | 7.0 (38) |
| Crush and snort the pill | 14.2 (77) |
| Other | 4.1 (22) |
| Average MDMA consumption per session |  |
| “I don’t know” | 15.5 (142) |
| <50mg | 7.2 (66) |
| 50-99mg | 8.8 (81) |
| 100mg | 9.0 (82) |
| 101-125mg | 6.8 (62) |
| 126-150mg | 9.2 (84) |
| 151-200mg | 13.0 (199) |
| 201-300mg | 16.3 (149) |
| 301-500mg | 8.3 (76) |
| 501-1000mg | 3.8 (35) |
| >1000mg | 2.0 (19) |
| Number of MDMA use occasions |  |
| 1 | 5.5 (50) |
| 2-5 | 15.5 (142) |
| 6-10 | 14.6 (134) |
| 11-20 | 16.3 (149) |
| 21-50 | 24.7 (226) |
| 51-100 | 10.5 (96) |
| >100 | 12.8 (118) |
| Frequency of MDMA use |  |
| More than once a week | 1.1 (10) |
| Weekly | 3.9 (36) |
| Fortnightly | 7.8 (71) |
| Monthly | 17.6 (161) |
| Every 2-3months | 28.5 (261) |
| Every 4-6months | 25.1 (230) |
| Once a year | 7.9 (72) |
| Less than once a year | 8.1 (74) |
| Decided to stop MDMA use | 19.1 (175) |
| Within the last year | 68.0 (119) |
| *Note.* Some response options regarding dose per session and number of use occasions have been collapsed together for ease of presentation. Some variables do not equal 100% due to rounding | |

| **Table S2**  *Reported Frequency of Acute MDMA effects (n* = 915*)* | | | | | |
| --- | --- | --- | --- | --- | --- |
| Item | Frequency % | | | | |
|  | Always | Most of the time | Sometimes | Almost Never | Never |
| Anxiety | 2.3 | 4.6 | 28.7 | 34.5 | 29.8 |
| Visual distortions | 3.1 | 14.3 | 39.0 | 24.2 | 19.5 |
| Heightened empathy/social connectedness | 47.3 | 33.9 | 13.0 | 2.7 | 3.1 |
| Greater energy | 52.9 | 36.6 | 7.9 | 1.5 | 1.1 |
| Heightened sexual arousal | 11.3 | 25.7 | 37.9 | 15.7 | 9.4 |
| Nausea | 1.7 | 5.6 | 27.3 | 36.0 | 29.4 |
| Confused thoughts | 1.5 | 8.4 | 34.5 | 33.7 | 21.9 |
| Euphoria | 42.1 | 39.6 | 11.5 | 3.6 | 3.3 |
| Greater appreciation of music | 54.0 | 32.8 | 9.9 | 1.6 | 1.6 |
| Difficulty urinating | 3.0 | 9.3 | 25.2 | 27.5 | 35.0 |
| Heightened appreciation of touch | 34.6 | 36.8 | 16.8 | 6.9 | 4.8 |
| Increased bowel movement | 4.7 | 8.6 | 28.4 | 31.1 | 27.1 |
| Tight/sore jaw | 19.8 | 36.0 | 31.7 | 7.4 | 5.1 |
| Reduced/loss of sexual interest | 3.4 | 7.1 | 29.1 | 31.7 | 28.7 |
| Dehydration | 11.3 | 26.4 | 38.7 | 15.7 | 7.9 |
| Intense positive emotion | 47.0 | 40.5 | 9.4 | 1.5 | 1.5 |
| Intense negative emotion | 1.2 | 3.0 | 14.6 | 36.6 | 44.6 |
| Have trouble standing or moving | 1.6 | 5.4 | 27.3 | 35.6 | 30.1 |
| Excessive sweating | 7.0 | 20.5 | 36.4 | 22.5 | 13.6 |
| Difficulty sleeping | 16.0 | 26.9 | 38.5 | 13.1 | 5.6 |

| **Table S3**  *Correlations between Harm Reduction Behaviours (n = 915)* | | | | | | | | | | | | | | | | | | |
| --- | --- | --- | --- | --- | --- | --- | --- | --- | --- | --- | --- | --- | --- | --- | --- | --- | --- | --- |
|  |  | 1 | 2 | 3 | 4 | 5 | 6 | 7 | 8 | 9 | 10 | 11 | 12 | 13 | 14 | 15 | 16 | |
| Source from a trusted person (1) | ρ | - | **.384^**^** | **-.390^**^** | .277^**^ | .270^**^ | .247^**^ | .223^**^ | .181^**^ | .193^**^ | **.529^**^** | .199^**^ | .287^**^ | .155^**^ | .224^**^ | .177^**^ | .165^**^ |  |
|  | Sig. | . | **<.001** | **<.001** | <.001 | <.001 | <.001 | <.001 | <.001 | <.001 | **<.001** | <.001 | <.001 | <.001 | <.001 | <.001 | <.001 |  |
| Planning use (2) | ρ | **.384^**^** | - | **-.339^**^** | .250^**^ | **.359^**^** | **.359^**^** | **.308^**^** | .228^**^ | .257^**^ | **.359^**^** | .228^**^ | **.314^**^** | .230^**^ | .167^**^ | .210^**^ | .089^**^ |  |
|  | Sig. | **<.001** | . | **<.001** | <.001 | **<.001** | **<.001** | **<.001** | <.001 | <.001 | **<.001** | <.001 | **<.001** | <.001 | <.001 | <.001 | .007 |  |
| Take MDMA offered by strangers (3) | ρ | **-.390^**^** | **-.339^**^** | - | **-.310^**^** | **-.383^**^** | **-.325^**^** | -.262^**^ | -.203^**^ | -.225^**^ | **-.346^**^** | -.143^**^ | -.216^**^ | -.161^**^ | -.186^**^ | -.143^**^ | -.092^**^ |  |
|  | Sig. | **<.001** | **<.001** | . | **<.001** | **<.001** | **<.001** | <.001 | <.001 | <.001 | **<.001** | <.001 | <.001 | <.001 | <.001 | <.001 | .005 |  |
| Not taking too much MDMA, staying in control (4) | ρ | .277^**^ | .250^**^ | **-.310^**^** | - | **.523^**^** | **.415^**^** | **.369^**^** | .212^**^ | **.367^**^** | .300^**^ | .211^**^ | **.303^**^** | .235^**^ | .192^**^ | .219^**^ | .132^**^ |  |
|  | Sig. | <.001 | <.001 | **<.001** | . | **<.001** | **<.001** | **<.001** | <.001 | **<.001** | <.001 | <.001 | **<.001** | <.001 | <.001 | <.001 | <.001 |  |
| Setting a limit of MDMA intake (5) | ρ | .270^**^ | **.359^**^** | **-.383^**^** | **.523^**^** | - | **.495^**^** | **.418^**^** | **.303^**^** | **.416^**^** | **.326^**^** | .221^**^ | .292^**^ | .275^**^ | .184^**^ | .244^**^ | .106^**^ |  |
|  | Sig. | <.001 | **<.001** | **<.001** | **<.001** | . | **<.001** | **<.001** | **<.001** | **<.001** | **<.001** | <.001 | <.001 | <.001 | <.001 | <.001 | .001 |  |
| Caution about mixing MDMA with other stimulants (6) | ρ | .247^**^ | **.359^**^** | **-.325^**^** | **.415^**^** | **.495^**^** | - | **.559^**^** | .291^**^ | **.370^**^** | **.334^**^** | .219^**^ | **.336^**^** | .279^**^ | .201^**^ | .287^**^ | .144^**^ |  |
|  | Sig. | <.001 | **<.001** | **<.001** | **<.001** | **<.001** | . | **<.001** | <.001 | **<.001** | **<.001** | <.001 | **<.001** | <.001 | <.001 | <.001 | <.001 |  |
| Caution about mixing MDMA with alcohol (7) | ρ | .223^**^ | **.308^**^** | -.262^**^ | **.369^**^** | **.418^**^** | **.559^**^** | - | **.319^**^** | **.326^**^** | **.345^**^** | .259^**^ | **.388^**^** | .255^**^ | .169^**^ | .254^**^ | .132^**^ |  |
|  | Sig. | <.001 | **<.001** | <.001 | **<.001** | **<.001** | **<.001** | . | **<.001** | **<.001** | **<.001** | <.001 | **<.001** | <.001 | <.001 | <.001 | <.001 |  |
| Spacing out MDMA use occasions well (8) | ρ | .181^**^ | .228^**^ | -.203^**^ | .212^**^ | **.303^**^** | .291^**^ | **.319^**^** | - | **.312^**^** | .209^**^ | .237^**^ | .234^**^ | .156^**^ | .128^**^ | .152^**^ | .059 |  |
|  | Sig. | <.001 | <.001 | <.001 | <.001 | **<.001** | <.001 | **<.001** | . | **<.001** | <.001 | <.001 | <.001 | <.001 | <.001 | <.001 | .073 |  |
| Waiting to feel effects before taking another dose (9) | ρ | .193^**^ | .257^**^ | -.225^**^ | **.367^**^** | **.416^**^** | **.370^**^** | **.326^**^** | **.312^**^** | - | **.301^**^** | .230^**^ | **.302^**^** | .292^**^ | .097^**^ | **.333^**^** | .132^**^ |  |
|  | Sig. | <.001 | <.001 | <.001 | **<.001** | **<.001** | **<.001** | **<.001** | **<.001** | . | **<.001** | <.001 | **<.001** | <.001 | .003 | **<.001** | <.001 |  |
| Confidence that substance is MDMA before consumption (10) | ρ | **.529^**^** | **.359^**^** | **-.346^**^** | .300^**^ | **.326^**^** | **.334^**^** | **.345^**^** | .209^**^ | **.301^**^** | - | .240^**^ | **.357^**^** | .272^**^ | .235^**^ | **.303^**^** | .158^**^ |  |
|  | Sig. | **<.001** | **<.001** | **<.001** | <.001 | **<.001** | **<.001** | **<.001** | <.001 | **<.001** | . | <.001 | **<.001** | <.001 | <.001 | **<.001** | <.001 |  |
| Get a good amount of rest following MDMA use (11) | ρ | .199^**^ | .228^**^ | -.143^**^ | .211^**^ | .221^**^ | .219^**^ | .259^**^ | .237^**^ | .230^**^ | .240^**^ | - | .447^**^ | .263^**^ | .097^**^ | .196^**^ | .107^**^ |  |
|  | Sig. | <.001 | <.001 | <.001 | <.001 | <.001 | <.001 | <.001 | <.001 | <.001 | <.001 | . | <.001 | <.001 | .003 | <.001 | .001 |  |
| Consumption a healthy amount of nutrients before and after MDMA use (12) | ρ | .287^**^ | **.314^**^** | -.216^**^ | **.303^**^** | .292^**^ | **.336^**^** | **.388^**^** | .234^**^ | **.302^**^** | **.357^**^** | **.447^**^** | - | **.319^**^** | .159^**^ | .259^**^ | .114^**^ |  |
|  | Sig. | <.001 | **<.001** | <.001 | **<.001** | <.001 | **<.001** | **<.001** | <.001 | **<.001** | **<.001** | **<.001** | . | **<.001** | <.001 | <.001 | <.001 |  |
| Seek out info about MDMA online (13) | ρ | .155^**^ | .230^**^ | -.161^**^ | .235^**^ | .275^**^ | .279^**^ | .255^**^ | .156^**^ | .292^**^ | .272^**^ | .263^**^ | **.319^**^** | - | .208^**^ | **.488^**^** | .055 |  |
|  | Sig. | <.001 | <.001 | <.001 | <.001 | <.001 | <.001 | <.001 | <.001 | <.001 | <.001 | <.001 | **<.001** | . | <.001 | **<.001** | .096 |  |
| Seek out info about MDMA from people I trust (14) | ρ | .224^**^ | .167^**^ | -.186^**^ | .192^**^ | .184^**^ | .201^**^ | .169^**^ | .128^**^ | .097^**^ | .235^**^ | .097^**^ | .159^**^ | .208^**^ | - | .132^**^ | **.593^**^** |  |
|  | Sig. | <.001 | <.001 | <.001 | <.001 | <.001 | <.001 | <.001 | <.001 | .003 | <.001 | .003 | <.001 | <.001 | . | <.001 | **<.001** |  |
| Seek out info about other drugs online (15) | ρ | .177^**^ | .210^**^ | -.143^**^ | .219^**^ | .244^**^ | .287^**^ | .254^**^ | .152^**^ | **.333^**^** | **.303^**^** | .196^**^ | .259^**^ | **.488^**^** | .132^**^ | - | .284^**^ |  |
|  | Sig. | <.001 | <.001 | <.001 | <.001 | <.001 | <.001 | <.001 | <.001 | **<.001** | **<.001** | <.001 | <.001 | **<.001** | <.001 | . | <.001 |  |
| Seek out info about other drugs from people I trust (16) | ρ | .165^**^ | .089^**^ | -.092^**^ | .132^**^ | .106^**^ | .144^**^ | .132^**^ | .059 | .132^**^ | .158^**^ | .107^**^ | .114^**^ | .055 | **.593^**^** | .284^**^ | - |  |
|  | Sig. | <.001 | .007 | .005 | <.001 | .001 | <.001 | <.001 | .073 | <.001 | <.001 | .001 | <.001 | .096 | **<.001** | <.001 | . |  |
| *Note.* Item names have been abbreviated for table clarity. | | | | | | | | | | | | | | | | | |  |

| **Table S4** *Percentage Agreement with Items Concerning Colorimetric Reagent Testing (n = 387)* | | | | | |
| --- | --- | --- | --- | --- | --- |
| Item | Agreement (%) | | | | |
|  | Strongly Agree | Agree | Neither agree nor disagree | Disagree | Strongly disagree |
| I always read the instructions provided or refresh my memory on how to use the test before I use it | 58.4 | 33.3 | 6.5 | 1.6 | 0.3 |
| I always observe the colour change carefully across time | 57.4 | 38.2 | 3.6 | 0.5 | 0.3 |
| When I use the test, I always use a large amount of my substance | 5.9 | 16.3 | 26.6 | 42.1 | 9.0 |
| I am always confident when interpreting the results of the test | 22.0 | 50.4 | 18.6 | 8.3 | 0.8 |
| I often worry that the sample I test may contain multiple active substances | 15.5 | 43.2 | 25.1 | 13.7 | 2.6 |
| I often worry that the sample I test may not contain any MDMA | 16.0 | 31.0 | 22.5 | 24.3 | 6.2 |
| *Note.* Each item is relevant to this testing method for different reasons. Items one, two and four are relevant to procedure and interpretation. Regarding item three, using a large amount of substance for the purposes of checking is not advised as this can impact colour change speed. With respect to item five, concern about the possibility of multiple active substance is important to consider as reagents are not able to be accurately interpreted when there are multiple active compounds present, as multiple colour changes can occur simultaneously. The same issue arises even if one is concerned about the lack of MDMA presence, although a complete lack of MDMA can be more informative as no colour change indicative of MDMA presence (or closely related compounds) will occur. | | | | | |

| **Table S5**  *Predictors of Past Use of a Colorimetric Reagent Test (n = 893)* | | |  |
| --- | --- | --- | --- |
| **Effect** | **OR (95% CI)** | **aOR (95% CI)** | |
| Age |  |  | |
| 18-21 vs 36+ | **0.53 (0.32-0.88)*** | 0.98 (0.51-1.90) | |
| 22-25 vs 36+ | 0.90 (0.54-1.50) | 1.06 (0.58-1.92) | |
| 26-29 vs 36+ | 1.40 (0.80-2.45) | 1.77 (0.93-3.37) | |
| 30-35 vs 36+ | 0.67 (0.37-1.23) | 0.78 (0.39-1.57) | |
| Female gender | **0.53 (0.40-70)***** | 0.87 (0.63-1.22) | |
| Māori ethnicity | 0.79 (0.54-1.17) |  | |
| Student | **0.64 (0.49-0.84)**** | 0.91 (0.61-1.36) | |
| Region of residence  (ref = Otago) |  |  | |
| Auckland | **2.25 (1.51-3.36)***** | 1.20 (0.72-2.00) | |
| Wellington | **2.01 (1.31-3.09)**** | 1.02 (0.59-1.75) | |
| Canterbury | **1.64 (1.12-2.40)*** | 1.13 (0.70-1.82) | |
| Other | 1.40 (0.93-2.12) | 0.86 (0.51-1.45) | |
| Frequency of MDMA use  (ref = once a year or less often) |  |  | |
| Weekly or more often | **2.34 (1.13-4.86)*** | 1.26 (0.54-2.95) | |
| Fortnightly | **4.36 (2.33-8.18)***** | 1.64 (0.79-3.42) | |
| Monthly | **3.95 (2.35-6.64)***** | 1.50 (0.82-2.74) | |
| Every 2-3 months | **4.03 (2.49-6.52)***** | 1.46 (0.84-2.55) | |
| Every 4-6 months | **2.60 (1.59-4.25)***** | 1.18 (0.67-2.08) | |
| Average dose per session (ref = 100mg) |  |  | |
| <50mg | **0.26 (0.11-0.59)**** | 0.43 (0.18-1.05) | |
| 51-99mg | 0.60 (0.30-1.18) | 0.72 (0.35-1.51) | |
| 101-150mg | **2.10 (1.19-3.69)*** | 1.56 (0.84-2.90) | |
| 151-250mg | **1.85 (1.05-3.26)**** | 1.81 (1.00-3.30) | |
| 251-500mg | **1.85 (1.05-3.26)*** | 1.40 (0.73-2.68) | |
| >500mg | 1.43 (0.70-2.90) | 1.60 (0.70-3.65) | |
| “I don’t know” | **0.29 (0.15-0.55)***** | **0.38 (0.19-0.77)**** | |
| Self-reported harm reduction knowledge (ref = know nothing) |  |  | |
| Know little | **2.64 (1.19-5.86)*** | **2.43 (1.05-5.66)*** | |
| Know moderate amount | **5.75 (2.67-12.36)***** | **4.18 (1.85-9.43)***** | |
| Know large amount | **12.42 (5.64-27.33)***** | **6.79 (2.92-15.83)***** | |
| MDMA harm | 1.28 (0.88-1.86) |  | |
| Non-MDMA harm | **2.15 (1.53-3.03)***** | **1.65 (1.11-2.44)*** | |
| Use of KYSNZ | **4.02 (2.93-5.51)***** | **2.58 (1.80-3.69)***** | |
| Use of colorimetric reagent test | - | - | |
| Meets dependence threshold | 1.41 (0.83-2.38) |  | |
| **Constant** |  | **0.09*** | |
| **Hosmer and Lemeshow Goodness-of-fit** |  | 0.648 | |
| *Note.* ^a^ < 0.10 * < 0.05, ** < 0.01, *** < 0.001. ref; reference category. All significant values are bold. | | |  |

| **Table S6**  *Reasons for not using the drug checking services of KnowYourStuffNZ (n = 583)* | |
| --- | --- |
| Item | Percentage (*n*) |
| It has never been available in my area | 32.76 (191) |
| It has never been at an event I have attended | 51.80 (302) |
| I am too nervous to approach/use the service | 10.98 (64) |
| It takes too long to get my drugs checked | 5.49 (32) |
| I have never been concerned with the content/quality/purity of my substances | 29.85 (174) |
| I don't want to give up some of my substance for checking | 6.35 (37) |
| The wait time was/is too long | 4.80 (28) |
| I can't be bothered going to get my drugs tested | 16.98 (99) |
| I am worried about being judged by the individuals working for KnowYourStuffNZ | 5.83 (34) |
| I am worried about being judged by other individuals using the drug checking service | 5.49 (32) |
| I am worried that if I am seen using the drug checking service, it may negatively impact my career | 11.15 (65) |
| Other | 17.50 (102) |
| *Note*. Multiple response items could be selected. | |

| **Table S7**  *Mann-Whitney U tests (MDMA harm vs. no MDMA harm) of MDMA-related Harm Reduction Behaviour Frequency* | | | | |
| --- | --- | --- | --- | --- |
| Item | Mean Ranks,  MDMA Harm | Mean Ranks, no MDMA Harm | Mann-Whitney U, *Z*, *p* |  |
| I source/buy my MDMA from a reliable and trusted person | 534.98 | 445.02 | **41517, -3.965, < 0.001** |  |
| I plan my use of MDMA in advance | 527.41 | 446.30 | **42515.5, -3496, < 0.001** |  |
| I take MDMA that is offered to me by strangers | 375.84 | 471.85 | **40833, -4.139, < 0.001** |  |
| When I take MDMA, I don’t take too much so that I am always in control and aware of my surroundings | 557.49 | 441.23 | **38545, -4.955, < 0.001** |  |
| I set a limit on how much MDMA I will take in a session and do not exceed that limit | 530.68 | 445.75 | **42084, -3.5335, < 0.001** |  |
| I am cautious about mixing MDMA with other stimulant drugs | 536.64 | 444.74 | **41297.5, -3.868, < 0.001** |  |
| I am cautious about mixing MDMA with alcohol | 531.49 | 445.61 | **41977.5, -3.531, < 0.001** |  |
| I space out the occasions where I use MDMA well | 516.26 | 448.18 | **43987.5, -2.846, 0.004** |  |
| When I take MDMA, I wait until I am feeling the effects before taking another dose | 499.83 | 450.96 | **46156, -2.125, 0.034** |  |
| I am confident that my MDMA is actually MDMA before I take it | 525.52 | 446.62 | **42765, -3.363, < 0.001** |  |
| Following MDMA use, I make sure to get a good amount of rest | 528.97 | 446.04 | **42310, -3.561, < 0.001** |  |
| Prior to and following MDMA use, I make sure to have a healthy amount of nutrients (food, supplements, water) | 568.16 | 439.43 | **37137.5, -5.439, < 0.001** |  |
| I seek out information about MDMA online when I want to know something | 477.52 | 454.71 | 49101.5, -0.986, 0.324 |  |
| I seek out information about MDMA from people I know and trust when I want to know something | 511.30 | 449.01 | **44642.5, -2.614, 0.009** |  |
| If a substance I thought was MDMA turns out to be another substance that I have not used, I seek out information about that substance online before deciding whether to consume it | 513.31 | 448.68 | **44377, -2.923, 0.003** |  |
| If a substance I thought was MDMA turns out to be another substance that I have not used, I seek out information about that substance from people I know and trust before deciding whether to consume it | 490.28 | 452.56 | 47417, -1.594, 0.111 |  |
| *Note*. Higher mean rank indicates lower frequency. Bold indicates a significant difference. | | | | |

| **Table S8**  *Mann-Whitney U tests (non-MDMA harm vs. no non-MDMA harm) of MDMA-related Harm Reduction Behaviour Frequency* | | | |  |
| --- | --- | --- | --- | --- |
| Item | Mean Ranks,  non-MDMA Harm | Mean Ranks, no non-MDMA Harm | Mann-Whitney U, *Z*, *p* | |
| I source/buy my MDMA from a reliable and trusted person | 515.78 | 444.91 | **53272, -3.450, < 0.001** | |
| I plan my use of MDMA in advance | 497.67 | 449.01 | **56332.5, -2.316, 0.021** | |
| I take MDMA that is offered to me by strangers | 399.60 | 471.23 | **53167, -3.410, < 0.001** | |
| When I take MDMA, I don’t take too much so that I am always in control and aware of my surroundings | 522.41 | 443.41 | **52151.5, -3.718, < 0.001** | |
| I set a limit on how much MDMA I will take in a session and do not exceed that limit | 503.78 | 447.63 | **55301, -2.581, 0.010** | |
| I am cautious about mixing MDMA with other stimulant drugs | 492.50 | 450.18 | **57206.5, -1.967, 0.049** | |
| I am cautious about mixing MDMA with alcohol | 499.74 | 448.54 | **55983.5, -2.325, 0.020** | |
| I space out the occasions where I use MDMA well | 499.55 | 448.59 | **56015, -2.353, 0.019** | |
| When I take MDMA, I wait until I am feeling the effects before taking another dose | 491.79 | 450.34 | **57326, -1.990, 0.047** | |
| I am confident that my MDMA is actually MDMA before I take it | 524.89 | 442.85 | **51732, -3.862, < 0.001** | |
| Following MDMA use, I make sure to get a good amount of rest | 485.01 | 451.88 | 58472, -1.725, 0.116 | |
| Prior to and following MDMA use, I make sure to have a healthy amount of nutrients (food, supplements, water) | 488.13 | 451.17 | 57944.5, -1.725, 0.085 | |
| I seek out information about MDMA online when I want to know something | 476.72 | 453.76 | 59873.5, -1.096, 0.273 | |
| I seek out information about MDMA from people I know and trust when I want to know something | 496.86 | 449.20 | **56470.5, -2.209, 0.027** | |
| If a substance I thought was MDMA turns out to be another substance that I have not used, I seek out information about that substance online before deciding whether to consume it | 467.37 | 455.88 | 61453.5, -0.574, 0.566 | |
| If a substance I thought was MDMA turns out to be another substance that I have not used, I seek out information about that substance from people I know and trust before deciding whether to consume it | 495.10 | 449.59 | **56766.5, -2.124, 0.034** | |
| *Note*. Higher mean rank indicates lower frequency. Bold indicates a significant difference. | | | |  |

**Figure 1**

*Non-MDMA Harm by MDMA Harm Status (n = 915)*


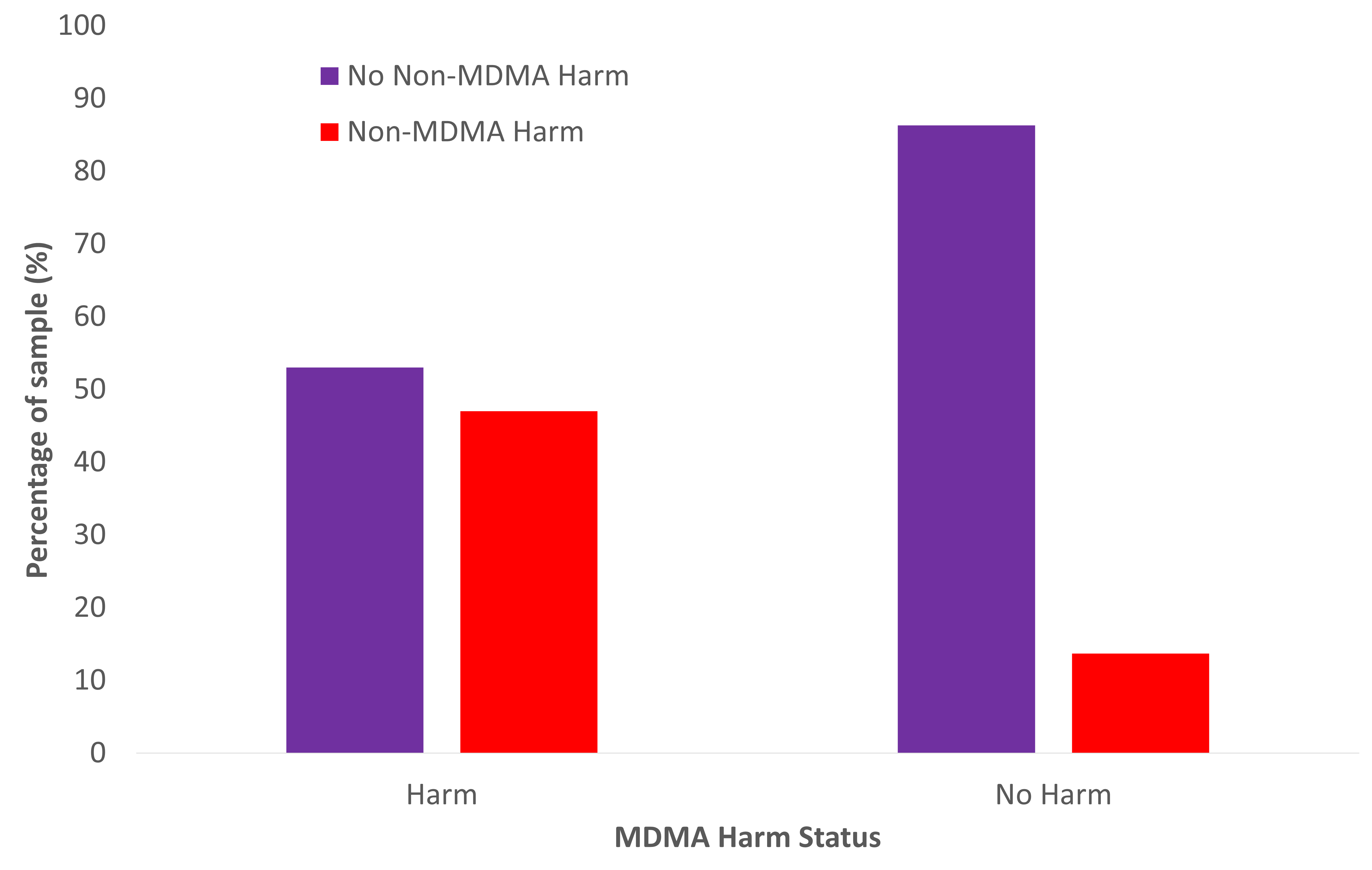

Supplement: Supplementary file 1 — Supplementary Material 1 [file 12954_2024_979_MOESM1_ESM.docx]
